# Phenotypic and genotypic characteristics of ESBL and AmpC producing organisms associated with bacteraemia in Ho Chi Minh City, Vietnam

**DOI:** 10.1186/s13756-017-0265-1

**Published:** 2017-10-16

**Authors:** Nguyen Phu Huong Lan, Nguyen Huu Hien, Tu Le Thi Phuong, Duy Pham Thanh, Nga Tran Vu Thieu, Dung Tran Thi Ngoc, Ha Thanh Tuyen, Phat Voong Vinh, Matthew J. Ellington, Guy E. Thwaites, Nguyen Van Vinh Chau, Stephen Baker, Christine J. Boinett

**Affiliations:** 1 The Hospital for Tropical Diseases, 764 Vo Van Kiet, Quan 5, Ho Chi Minh City, Vietnam; 2 Oxford University Clinical Research Unit, The Hospital for Tropical Diseases, Wellcome Trust Major Overseas Programme, Ho Chi Minh City, Vietnam; 3 Centre for Tropical Medicine and Global Health, Nuffield Department of Clinical Medicine, Oxford University, Oxford, UK; 4 Antimicrobial Resistance and Healthcare Associated Infection Unit, National Infection Service, Public Health England, Public Health England, London, UK; 5 The Department of Medicine, Cambridge University, Cambridge, UK

**Keywords:** ESBL, AmpC, Bacteraemia, Antimicrobial resistance

## Abstract

**Background:**

Broad-spectrum antimicrobials are commonly used as empirical therapy for infections of presumed bacterial origin. Increasing resistance to these antimicrobial agents has prompted the need for alternative therapies and more effective surveillance. Better surveillance leads to more informed and improved delivery of therapeutic interventions, potentially leading to better treatment outcomes.

**Methods:**

We screened 1017 Gram negative bacteria (excluding *Pseudomonas* spp. and *Acinetobacter* spp.) isolated between 2011 and 2013 from positive blood cultures for susceptibility against third generation cephalosporins, ESBL and/or AmpC production, and associated ESBL/AmpC genes, at the Hospital for Tropical Diseases in Ho Chi Minh City.

**Results:**

Phenotypic screening found that 304/1017 (30%) organisms were resistance to third generation cephalosporins; 172/1017 (16.9%) of isolates exhibited ESBL activity, 6.2% (63/1017) had AmpC activity, and 0.5% (5/1017) had both ESBL and AmpC activity. *E. coli* and *Aeromonas* spp. were the most common organisms associated with ESBL and AmpC phenotypes, respectively*.* Nearly half of the AmpC producers harboured an ESBL gene. There was no significant difference (*p* > 0.05) between the antimicrobial resistance phenotypes of the organisms associated with community and hospital-acquired infections.

**Conclusion:**

AmpC and ESBL producing organisms were commonly associated with bloodstream infections in this setting, with antimicrobial resistant organisms being equally distributed between infections originating from the community and healthcare settings. *Aeromonas* spp., which was associated with bloodstream infections in cirrhotic/hepatitis patients, were the most abundant AmpC producing organism. We conclude that empirical monotherapy with third generation cephalosporins may not be optimum in this setting.

**Electronic supplementary material:**

The online version of this article (10.1186/s13756-017-0265-1) contains supplementary material, which is available to authorized users.

## Background

Antimicrobial resistance (AMR) is an increasing problem in global health. Resistance against antimicrobials used for empirical therapy of invasive infections is increasing at an alarming rate and the provision of effective antimicrobials improves the likelihood of a better clinical outcome. To limit the possibility of poor outcomes, broad-spectrum antimicrobials, such as third generation cephalosporins, have become the most commonly used empirical drugs to treat non-specific febrile diseases but this has come at the cost of increased resistance to these drugs. The situation has been recognised internationally and the WHO have identified seven bacteria as the most important cause of AMR infections in hospitals and the community. This list includes *Escherichia coli* and *Klebsiella pneumoniae*, both commonly found to be resistant to third generation cephalosporins [1]. Resistance to third generation cephalosporins can be mediated by a class of serine hydrolases known commonly as the extended-spectrum β-lactamases (ESBLs), which act by cleaving the β-lactam ring, thereby rendering the drug inactive [2]. With the increasing threat of treatment failure, monitoring ESBL producing organisms in sentinel locations is imperative for surveillance and appropriate treatment strategies.

Routine susceptibility testing is usually capable of detecting the presence of ESBL activity, however false positives can occur. This lack of sensitivity is associated with plasmid-mediated ampicillinases known as AmpC. AmpC genes confer resistance to many β-lactams and β-lactam/β-lactamase inhibitor combinations, the latter of which render ESBL producing bacteria susceptible. AmpC genes are generally located chromosomally, however plasmid associated variants of the enzymes have become increasingly recognised. This observation is significant as these ampicillinases can be easily disseminated by horizontal gene transfer [3]. There are currently no standardised Clinical and Laboratory Standards Institute (CLSI) guidelines for AmpC detection, although several methods have been proposed to aid in the accurate detection of AmpC β-lactamases [4–6]. These phenotypic tests primarily use a cephalosporin with a β-lactamase inhibitor (e.g. clavulanate) or a non-β-lactamase inhibitor (e.g. boronic acid) and can be combined with molecular detection of the AmpC β-lactamases using multiplex PCR [7]. However, due to over-expression of AmpC genes, bacteria carrying an ESBL gene may test negative for ESBL production, resulting in misinterpretations of phenotypic AmpC methods [8–10], which can have significant consequences for patient care. The accurate testing and interpretation of AmpC and ESBL activity is vital for healthcare professionals to provide effective and appropriate treatment management. Here, we aimed to assess the distribution of AmpC and ESBL genes in organisms associated with bloodstream infections at the Hospital for Tropical Diseases (HTD) in Ho Chi Minh City (HCMC), Vietnam. Our data highlights the need for routine AmpC and ESBL gene surveillance in hospitals in low-middle income countries to ensure effective treatment and report on the incidence of drug resistant bacteraemia.

## Methods

### Ethics statement

Ethical approval for this study was provided by the ethical review board of HTD in Ho Chi Minh City (HCMC), Vietnam.

### Study setting and patient population

HTD is a 550-bed infectious disease hospital that serves as a primary and secondary facility for the surrounding local population in HCMC and a tertiary referral centre for infectious diseases for the 17 southern provinces of the country. Neonates, patients without infectious diseases, including those with surgical requirements, tuberculosis, cancer, primary haematological disorders or immunosuppression (other than HIV) are referred to other hospitals in the city.

Blood cultures were performed for patients in whom an infection was suspected on the basis of a fever (> 38 °C) or who had evidence of sepsis on the basis of the presence of two or more of the following features: fever (> 38 °C) or low temperature (< 36 °C); tachycardia (exact level according to age); tachypnea (exact level according to age); an elevated white cell count (> 12,000 cells/mm^3^) or depressed white cell count (< 4000 cells/mm^3^). There was no systematic change in the application of these criteria during the time course of the study. All data originating from consecutive patients admitted to the hospital who had a blood culture performed for suspected bloodstream infection between January 1st 2011 and December 31st 2013 were included in this retrospective study. Routinely, a member of the hospital staff recorded the date of blood draw, the patient’s age, sex, and suspected diagnosis, the number of blood culture bottles inoculated, the result of the culture (whether positive or negative) and the susceptibility of the isolate to commonly used antimicrobial agents. These are the source data for this study. For the purposes of these analyses blood samples taken for culture > 48 h after admission to HTD were classified as a Hospital Acquired Infection (HAI) and samples taken within 48 h of admission to HTD were classed as a Community Acquired Infection (CAI).

### Blood culturing and bacterial identification

Venous blood cultures of 8–15 mL from adults and 2–5 mL of venous blood from infants and children were routinely obtained and inoculated into BACTEC*plus* aerobic bottles (Becton Dickenson, USA). Inoculated BACTEC bottles were incubated at 37 °C in a BACTEC 9050 automated analyser for up to 5 days and sub-cultured when the machine indicated a positive signal. All sub-cultures were plated onto fresh sheep blood agar (Oxoid Unipath, Basingstoke, United Kingdom). Plates were incubated at 37 °C in air for 5 days and organisms were subsequently identified by standard methods including API20E and API20NE identification kits (Bio-Mérieux, France). *Staphylococcus aureus* ATCC 29213 and *Pseudomonas aeruginosa* ATCC 27853 were used as controls.

### Antimicrobial susceptibility testing

The susceptibility to relevant antimicrobial agents was determined by the modified Bauer-Kirby disc diffusion method. Enterobacteriaceae were tested with discs containing chloramphenicol (30 μg), ampicillin (10 μg), co-trimoxazole (trimethoprim 1.25 μg/sulfamethoxazole 23.75 μg), ceftriaxone (30 μg), ofloxacin (5 μg) and gentamicin (10 μg). The breakpoint zone sizes were interpreted according to CLSI guidelines [11].

The double disk diffusion method [12] was used to identify ESBL activity. This method was performed using a combination of cefepime (30 μg), ceftazidime (30 μg), ceftriaxone (30 μg), Amoxicillin (20 μg)/clavulanate (10 μg). ESBL producers were identified by reduced zone sizes to third-generation cephalosporins (ceftazidime and ceftriaxone), and expansion of these zones in the presence of an inhibitor (clavulanate). Confirmatory tests for ESBL producers were performed using oxyamino-cephalosporins/β-lactam inhibitor combinations, namely cefotaxime (30 μg)/clavulanate (10 μg) and ceftazidime (30 μg)/clavulanate (10 μg) (Additional file 1: Figure S1B). Zone sizes were measured and interpreted according to the CLSI guidelines [11].

Phenotypic AmpC activity was detected using two different tests. First, we measured zone sizes using a combination of cefoxitin and an alternative third- and fourth-generation cephalosporin, where an AmpC positive organism would be resistant to cefoxitin and exhibit reduced susceptibility to the alternative third generation cephalosporin (ceftriaxone 30 μg) and complete susceptibility to the fourth generation cephalosporin (cefepime). Secondly, we determined inducible AmpC phenotypes by assessing reduced zone sizes against a third generation cephalosporin (ceftriaxone 30 μg or ceftazidime 30 μg) in the presence of imipenem (30 μg) as an inducing substrate (Additional file 1: Figure S1A) [13, 14].

### Genotypic screening of ESBL and AmpC genes

Blood isolates collected from the same patient at two different time-points with the same phenotypic AMR profile were denoted as duplicates and only one was subsequently selected for further PCR analysis. If the AMR profile of the two isolates selected differed, both were selected for PCR analysis. Multiplex PCR reactions were used to detect ESBL (*bla*_CTX-M_ subtypes) [15] and AmpC [7] genes. Other β-lactamase genes, *bla*_TEM_ [16], *bla*_OXA_ [17]_,_ and *bla*_SHV_ [16] were also detected by multiplex PCR using the following cycling conditions, initial denaturation step at 95 °C for 15 min, followed by 25 cycles of DNA denaturation at 94 °C for 30 s, primer annealing at 57 °C for 40 s, and primer extension at 72 °C for 30 s. After the last cycle, a final extension step at 72 °C for 10 min was added. PCR amplicons were examined by agarose gel (BioRad) electrophoresis made up to a concentration of 1.5% (*w*/*v*). The primers used in this study are shown in Additional file 2: Table S1.

All positive ESBL (*bla*_CTX-M_) and AmpC (*bla*_CIT_) PCR amplicons were sequenced to further subtype these genes. DNA was extracted using the Agencourt AMPure XP PCR purification system (Beckman Coulter) and sequenced using the BigDye Terminator v3.1 cycle sequencing kit (Applied biosystems) on the 3130 genetic analyser (Applied biosystems). DNA sequences were compared against the National Centre of Biotechnology Information (NCBI) GenBank sequence database using the BLASTn algorithm and gene variants were subsequently deduced based on sequence similarity.

### Statistical analysis

Statistical analysis was performed in R. Comparisons between HAI and CAI antimicrobial resistance instance rates was assessed using the Fisher’s exact test (two-tailed). A *p*-value < 0.05 was considered statistically significant.

## Results

### Gram-negative organisms with reduced susceptibility against third generation cephalosporins isolated from bloodstream infections at the Hospital for Tropical Diseases

Between January 2011 and December 2013, 1690 non-contaminant bacteria were isolated from the blood of febrile patients attending HTD in HCMC. The overall isolation rate from blood during this period was 5.6% (1690/30,185); 1017 (60%) of these were Gram-negative (excluding *Pseudomonas* spp. and *Acinetobacter* spp.) (Table 1). In > 70% (783/1017) of cases, a member of the Enterobacteriaceae was found to be the causative agent, with *E. coli* accounting for 56% (445/783) of these infections. Nearly half of these isolates were resistant to at least three different antimicrobial classes, including third generation cephalosporins. *Aeromonas* spp. was the most abundant (59/234; 5.8%) non-Enterobacteriaceae Gram-negative genus isolated from the blood cultures, with 27/59 (46%) of the isolated *Aeromonas* spp. being resistant to a third generation cephalosporin.

**Table 1:** Summary of Gram-negative microorganisms isolated from blood cultures during the period of 2011–2013

				Resistance phenotype												
Species	CAI (*n* = 876)	HAI (*n* = 141)	Total (*n* = 1017)	amk	amc	caz	cip	cro	etp	fep	imp	mem	ofx	sxt	tcc	tzp
Enterobacteriaceae (*n* = 783)																
*Edwardsiella* spp.	2	0	2 (0.2%)	–	–	–	–	–	–	–	–	–	1	1	–	–
*Enterobacter* spp.	9	4	13 (1.3%)	–	8	8	1	10	–	1	–	–	1	4	5	1
***E. coli***	**392**	**53**	**445 (43.8%)**	2	80	201	196	203	5	179	1	1	200	298	262	24
***Klebsiella*** **spp.**	**127**	**27**	**154 (15.1%)**	1	15	23	19	24	3	18	2	3	19	51	30	8
*Morganella morganii*	2	1	3 (0.3%)	–	3	2	–	3	–	–	1	–	–	1	–	–
*Proteus mirabilis*	2	6	8 (0.8%)	–	–	1	3	1	–	1	–	–	3	5	1	–
*Providencia* spp.	1	1	2 (0.2%)	–	1	–	–	–	–	–	–	–	–	–	–	–
**Non-typhoidal** ***Salmonella***	102	9	**111 (10.9%)**	–	–	1	3	1	–	1	–	–	3	29	28	–
*Salmonella* Typhi	40	4	44 (4.3%)	–	–	–	–	–	–	–	–	–	–	11	–	–
*Serratia marcescens*	0	1	1 (0.1%)	–	1	–	–	1	–	–	–	–	–	–	–	–
Non-Enterobacteriaceae (*n* = 234)																
***Aeromonas*** **spp.**	**51**	**8**	**59 (5.8%)**	–	24	23	–	27	–	–	–	–	1	9	20	–
*Abiotrophia* spp.	1	0	1 (0.1%)	–	–	–	–	–	–	–	–	–	–	1	–	–
*Archromobacter xylosoxidans*	1	1	2 (0.2%)	1	–	1	1	2	–	1	–	–	–	1	1	–
*Alcaligenes* spp.	8	3	11 (1.1%)	4	2	1	1	3	–	4	–	–	1	1	1	–
*Brevundimonas vesicularis*	2	0	2 (0.2%)	No information available												
***Burkhoderia cepacia***	**28**	**8**	**36 (3.5%)**	–	–	1	–	–	–	–	–	–		1	–	–
***Burkhoderia pseudomallei***	**51**	**0**	**51 (5.0%)**	–	–	–	–	–	–	–	–	–	–	–	–	–
*Campylobacter* spp.	13	0	13 (1.3%)	–	–	–	8	–	–	–	–	–	10	–	–	–
*Chryseomonas* spp.	2	2	4 (0.4%)	3	2	1	2	2	–	2	3	3	2	2	2	1
*Comamonas* spp.	1	1	2 (0.2%)	No information available												
*Gemella* spp.	1	0	1 (0.1%)	No information available												
*Haemophilus* spp.	9	2	11 (1.1%)	–	1	–	5	1	–	–	–	–	–	8	–	–
*Moraxella* spp.	1	1	2 (0.2%)	–	–	–	–	–	–	–	–	–	–	–	–	–
*Neisseria meningitidis*	5	0	5 (0.5%)	–	–	–	–	–	–	–	–	–	–	–	–	–
*Pasteurella* spp.	6	0	6 (0.6%)	1	–	–	–	1	–	1	–	–	–	–	–	–
*Psychrobacter* spp.	2	0	2 (0.2%)	No information available												
*Ralstonia pickettii*	4	2	6 (0.6%)	4	1	1	–	–	–	2	1	2	–	1	3	1
*Roseomonas* spp.	2	0	2 (0.2%)	1	–	–	–	–	–	–	–	–	–	–	–	–
*Sphingomonas* spp.	2	1	3 (0.3%)	–	1	2	–	–	–	2	–	–	–	–	–	1
*Stenotrophomonas maltophilia*	6	5	11 (1.1%)	–	–	7	–	–	–	–	–	–	–	2	6	–
*Vibrio* spp.	3	1	4 (0.4%)	–	–	–	–	–	–	–	–	–	–	1	–	–

Highlighted in bold are the top three Gram-negative microorganisms (Enterobacteriaceae and non-Enterobacteriaceae) isolated from blood cultures

*Abbreviations*: *amk* amikacin, *amc* amoxicillin-clavulanic acid, *caz* ceftazidime, *cip* ciprofloxacin, *cro* ceftriaxone, *etp* ertapenem, *fep* cefepime, *imp* imipenem, *mem* meropenem, *ofx* ofloxacin, *sxt* trimethoprim-sulfamethoxazole, *tcc* ticarcillin-clavulanic acid, *tzp* piperacillin-tazobactam

Ceftriaxone (or an alternative parenteral third generation cephalosporin) was (and remains) the empirical therapy for suspected bacteraemia/sepsis in this healthcare facility. Therefore, all Gram-negative organisms isolated from blood were routinely screened for susceptibility against various third generation cephalosporins. In total, nearly a third (304/1017; 30%) were found to exhibit reduced susceptibility against third generation cephalosporins, with > 90% (*n* = 280) of these exhibiting resistance to ceftriaxone and nearly 3% (*n* = 8) being intermediate according to current CLSI susceptibility breakpoints [11].

**Fig. 1:**
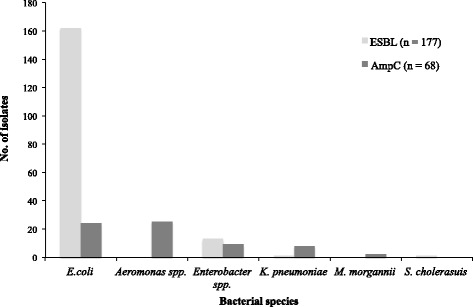
The distribution of ESBL or AmpC producing bacteria isolated during the period of 2011–2013. Bar chart indicates the number and type of ESBL or AmpC producing bacteria isolated

The 280 non-duplicate organisms exhibiting resistance to ceftriaxone were subjected to phenotypic screening using the double disk diffusion method to detect ESBL activity, and to identify a zone of reduced inhibition between the third generation cephalosporin and imipenem, indicative of AmpC activity (Additional file 1: Figure S1). In total 172/1017 (16.9%) isolates were phenotypically ESBL positive; 63/1017 (6.2%) were phenotypically AmpC positive and 5/1017 (0.5%) exhibited phenotypic evidence of both ESBL and AmpC activity. *E. coli* was the most common bacterial species exhibiting ESBL activity only, or both ESBL and AmpC activity, accounting for > 90% (162/177) of this group of ceftriaxone resistant organisms (Fig. 1). Approximately 40% (25/63) of the AmpC producing organisms were *Aeromonas* spp.; *E. coli* was the second most abundant species amongst the AmpC producers with 23/68 (> 30%) identified. The remaining organisms exhibiting AmpC activity belonged to a range of species, including *Enterobacter* spp. (9/68), *K. pneumoniae* (8/68), and *Morganella morganii* (2/68).

### Molecular analysis of ESBL and AmpC genes conferring resistance to third generation cephalosporins

PCR amplification was performed on nucleic acid extracted from the 177 ESBL (172 ESBL and 5 producing both ESBL and AmpC) exhibiting phenotypic ESBL activity to identify the four most common ESBL gene groups (*bla*_TEM_, *bla*_SHV_, *bla*_OXA,_ and *bla*_CTX-M_). These PCR amplifications demonstrated that *bla*_CTX-M_ was the most prevalent ESBL gene family in these organisms, testing positive in 168/177 (95%) nucleic acid extractions; *E. coli* was the most frequently identified organism harbouring the *bla*_CTX-M_ gene (Table 2). Sixty per cent (106/177) of these isolates had multiple ESBL genes, the most frequent combinations were *bla*_CTX-M_ with *bla*_TEM_ (summarised in Additional file 3: Table S2). Subsequent sequencing of the *bla*_CTX-M_ PCR amplicons revealed that *bla*_CTX-M-15_ (*n* = 84) was the most common ESBL gene subtype. Other ESBL gene subtypes detected included *bla*_CTX-14_ (*n* = 40), *bla*_CTX-24_ (*n* = 6), *bla*_CTX-27_ (*n* = 48), and *bla*_CTX-55_ (*n* = 12) (Additional file 4: Table S3). Of the five isolates exhibiting both an ESBL and an AmpC phenotype, only two had genetic determinants that were associated with an AmpC phenotype, namely, *bla*_CIT_ (CMY-42), and *bla*_EBC_. Two *bla*_CTX-M_ variants (*bla*_CTX-M-15_ and *bla*_CTX-M-27_) were identified in these organisms as the genes most likely to confer resistance against third generation cephalosporins (Additional file 4: Table S3).

**Table 2:** The distribution of β-lactamase genes from the 177 ESBLs producing bacteria identified

Gene	*E. coli*	*Klebsiella* spp*.*	*Enterobacter cloacae*	*Salmonella cholerasuis*	Total^a^
*bla*_CTX-M_	157	9	1	1	168 (95%)
*bla*_TEM_	70	7	1	1	79 (45%)
*bla*_OXA_	33	2	1	–	36 (20%)
*bla*_SHV_	1	4	–	–	5 (3%)
Not detected	3	1	–	–	4 (2%)

^a^The proportion of bacterial species harbouring a particular ESBL gene is expressed as a percentage of the total bacteria possessing an ESBL phenotype (*n* = 177)

PCR amplification of nucleic acid extracted from the 68 organisms (63 AmpC and 5 isolates producing both ESBL and AmpC) with AmpC activity found that *bla*_CIT_ was the most common gene associated with this phenotype; this variant was detected in 19/68 (28%) of the isolates (Table 3). Sequencing of the AmpC amplicons determined that approximately half (11/19; 58%) of the isolates harboured the *bla*_CMY-2_ subtype; the remainder (8/19; 42%) carried the *bla*_CMY-42_ gene. Notably, more than half (37/68; 54%) of the organisms conferring an AmpC phenotype did not generate a PCR amplicon for any of the six AmpC genetic markers that were screened. The majority of the organisms exhibiting AmpC activity but not generating any detectable PCR products were *Aeromonas* spp. The remainder of the AmpC producing organisms harboured either *bla*_DHA_ (9/68; 13%) or *bla*_EBC_ (3/68; 4%) genes. Almost half (32/68; 47%) of the AmpC producing isolates also generated a PCR amplicon for an ESBL gene. The most commonly detected ESBL gene in these phenotypic AmpC organisms was the *bla*_TEM_ gene, found in 17/32 (53%) of these isolates (Table 4). Three *E. coli* isolates harboured three different β-lactamase genes, *bla*_CTX-M_, *bla*_TEM_, and *bla*_OXA_.

**Table 3:** The distribution of β-lactamase genes from the AmpC producing bacteria identified

Gene	*Aeromonas* spp*.*	*E. coli*	*Klebsiella* spp*.*	*Enterobacter* spp*.*	*M. morganii*	Total^a^
*bla*_CIT_	–	18	–	1	–	19 (28%)
*Bla*_DHA_	–	1	6	–	2	9 (13%)
*Bla*_EBC_	–	–	–	3	–	3 (4%)
Not detected	25	5	2	5	–	37 (54%)

^a^The proportion of bacterial species harbouring a particular AmpC gene is expressed as a percentage of the total bacteria possessing an AmpC phenotype (*n* = 68)

**Table 4:** The distribution of the 68 AmpC producing bacteria harbouring an additional ESBL gene

Gene	*Aeromonas* spp.	*E. coli*	*Klebsiella* spp.	*Enterobacter* spp.	*M. morganii*	Total^a^
*bla*_CTX-M_		3				3 (4%)
*bla*_TEM_	–	15	–	1	1	17 (25%)
*bla*_SHV_	–	–	4	1	–	5 (7%)
*bla*_OXA_	–	2	–	–	–	2 (3%)
*bla*_OXA_ & *bla*_TEM_	–	1	1	–	–	2 (3%)
*bla*_OXA_, *bla*_TEM_ *& bla*_CTX-M_	–	1	1	1	–	3 (4%)
Not detected^b^	25	2	2	6	1	36 (53%)

^a^The proportion of all bacterial species with AmpC phenotype (*n* = 68) gene is expressed as a percentage of the total bacteria possessing harbouring an additional ESBL gene

^b^These samples had an ESBLs phenotype but no detectable ESBL gene

### Hospital and community-acquired third-generation cephalosporin resistant infections

**Fig. 2:**
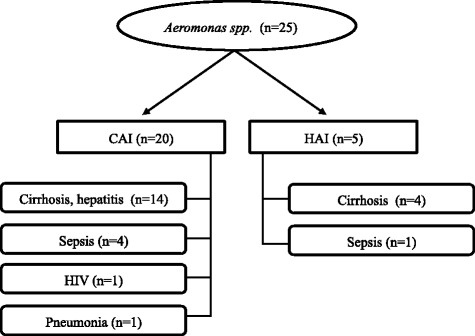
The distribution of bacteremia infections where Aeromonas spp. was isolated from community (CAI) and hospital (HAI) patients. From 63 AmpC producing bacteria isolates, 25 were found to be *Aeromonas* spp. Indicated below the CAI/HAI designation, are the infections the patietns presented at the point of sample collection. We found cirrhosis/hepatitis to be significantly asosiated (*p* < 0.001) infection by *Aeromonas* spp.

*E.coli* and *Aeromonas* spp*.* were the most common bacterial species identified as being ESBL or AmpC producing, respectively. Ninety-seven per cent (157/162) of the *E. coli* isolates with an ESBL phenotype harboured a *bla*_CTX-M_ gene. Of the 162 phenotypically identified as ESBL producers, 140 were associated with a CAI and 22 were associated with a HAI. Hypothesising that organisms associated with HAI would have a broader AMR range, we compared the antimicrobial susceptibility profiles of organisms associated with CAIs and HAIs. However, after investigating susceptibility against a range of different antimicrobial classes in the ESBL producing organisms, we found no significant difference between the antimicrobial susceptibility profiles of organisms associated with CAI or HAI (*p* > 0.05; Fisher’s exact test) (Additional file 5: Figure S2A). We additionally compared antimicrobial susceptibility phenotypes between *E. coli* associated with CAI and HAI for the isolates that were phenotypically AmpC positive. There was no significant difference in the antimicrobial susceptibility profiles between these groups (*p* > 0.05; Fisher’s exact test) (Additional file 5: Figure S2B). Lastly, we investigated the clinical source of the AmpC producing *Aeromonas* spp. in CAI and HAI. We found that 72% (18/25) of the AmpC expressing *Aeromonas* spp. were associated with patients with underlying liver cirrhosis or hepatitis (Fig. 2).

## Discussion

With the ever-increasing reports of ESBL producing Gram-negative bacteria in clinical settings, identifying these organisms is imperative for disease monitoring and the provision of efficacious treatments. Although antimicrobial susceptibility profiling is still the most commonly used method, interpretation of AmpC production data can sometimes prove problematic and may lead to under-reporting or misdiagnosis as an ESBL phenotype [18]. This scenario could potentially lead to treatment failure due to the use of alternative fourth-generation cephalosporins or carbapenems. Here, we aimed to investigate the prevalence and diversity of ESBL and AmpC producing isolates organisms isolated from bloodstream infections over a 3-year period at a major infectious disease hospital in Vietnam.

CTX-M type ESBLs are the most prevalent ESBL enzymes to be reported, with > 170 different subtypes assigned [19, 20]. Our study identified these enzymes to be largely associated with *E. coli* isolates originating from the community. This is not surprising, as a previous studies from the same region found that healthy individuals had a high carriage rate of ESBL producing *Enterobacteriaceae* in their gastrointestinal microbiota [21]. Furthermore, we found no statistical significance in overall AMR carriage between CAI and HAI in *E. coli* harbouring an ESBL or AmpC phenotype. This observation is important and suggests equilibrium between circulating AMR organisms with the potential to cause disease in the community and the clinical setting in this location. We identified an additional ESBL gene in approximately half of the AmpC producing organisms. The presence of a co-produced ESBL may affect the interpretation of a phenotypic AmpC detection test [8, 18, 22], therefore the treatment of infections associated with suspected AmpC producing organisms with cefepime requires additional microbiological assessment for production of an ESBL.

No plasmid associated resistance genes were detected for the most prevalent AmpC producing organism identified in this study, *Aeromonas* spp*.* We speculate that the AmpC phenotype in these organisms was mediated by a chromosomally located AmpC gene [4, 15]. Notably, when assessing the clinical presentations associated with the AmpC producing *Aeromonas* spp., these organisms were found to be associated with cirrhosis/hepatitis. *Aeromonas* spp. are commonly identified as the agent of spontaneous bacterial peritonitis with bacteraemia in patients with liver cirrhosis, and commonly has a higher mortality rate than infections caused by alternative pathogens [23, 24]. The high prevalence of *Aeromonas* spp. mediated cirrhotic infections suggests the spread of this bacterial organism within immunocompromised chronic liver patients in HCMC, a potential association that requires longitudinal surveillance.

## Conclusions

We observed a high prevalence of AmpC and ESBL expressing organisms associated with CAI and HAI bloodstream infections hospitalised patients in HCMC. We additionally report that AmpC producing *Aeromonas* spp*.* are potentially associated with bacteraemia in patients with underlying cirrhosis/hepatitis. With febrile disease presentations, it is routine clinical practice in this location to prescribe a broad-spectrum antimicrobial, generally a third-generation cephalosporin, to increase the likelihood of a better clinical outcome [25, 26]. However, routine hospital data suggests that there has been in increase in the proportion of ESBL producing *E. coli* isolated from blood from 45 to > 60% between 2010 and 2014. Taken together we suggest that the use of third generation cephalosporins in monotherapy may not be an optimum approach and alternatives should be assessed carefully when there is a clinical suspicion of bacteraemia, irrespective of whether this is a suspected CAI or HAI.

## Additional files

Additional file 1: Figure S1. Representative results of the double disk diffusion test (A) for ESBL production and the AmpC disk test (B). Abbreviations. AMC: amoxicillin, CTX: cefotaxime, CAZ: ceftazidime, CRO: ceftriaxone, CLA: clavulanate, FEP: cefepime, FOX: cefoxitin, IPM: imipenem. (PDF 281 kb)

Additional file 2: Table S1. The AmpC and ESBL primers used in this study. (DOCX 100 kb)

Additional file 3: Table S2. Summary of ESBL genes identified in 177 ESBL producing isolates. (DOCX 49 kb)

Additional file 4: Table S3. The genetic variants of the 177 ESBL producers. Key: * indicates the isolates that produce AmpC and ESBL phenotype: E063, E080, E081, E113 and E126 . ^#^denotes ESBL encoding isolates that encoded an AmpC gene but did not have an AmpC phenotype. The *bla*_EBC_ variants includes (MIR-1 and ACT-1) (XLSX 54 kb)

Additional file 5: Figure S2. Comparison of antimicrobial susceptibility profiles between CAI and HAI of the 177 ESBL (A) and 63 AmpC (B) producing *E. coli.* (A) The AMR phenotype of the ESBL producing *E. coli* (A) (*n* = 162) from CAI (*n* = 140) or HAI (*n* = 22) was scored for resistance to each drug and this was expressed as a percentage relative to the number of organisms in each group (CAI or HAI). (B) The AMR phenotype of the AmpC producing *E. coli* (*n* = 17) from CAI (*n* = 13) or HAI (*n* = 4) was scored for resistance to each drug and this was expressed as a percentage relative to the number of organisms in each group (CAI or HAI). No significant differences (*p* > 0.05) were found in antimicrobial resistance phenotype between CAI and HAI in either ESBL or AmpC producers. (PDF 47 kb)

## Abbreviations

AmpC: Ampicillin-hydrolyzing cephalosporinase
AMR: Antimicrobial resistance
CAI: Community acquired infections
CSF: Cerebral spinal fluid
ESBLs: Extended-spectrum β-lactamases
HAI: Hospital acquired infections
HCMC: Ho Chi Minh City
HTD: Hospital of tropical diseases

## References

[1] WHO. Antimicrobial Resistance: World Heal Organ; 2013. http://www.who.int/mediacentre/factsheets/fs194/en/

[2] Bradford P (2001). Extended spectrum beta-lactamase in the 21 century: characterization, epidemiology, and detection of this important resistant threat. Clin Microbiol Rev.

[3] Doi Y, Paterson DL (2007). Detection of plasmid-mediated class C β-lactamases. Int J Infect Dis.

[4] Jacoby GA (2009). AmpC β-Lactamases. Clin Microbiol Rev.

[5] Gupta G, Tak V, Mathur P (2014). Detection of AmpC β Lactamases in gram-negative bacteria. J Lab Physicians.

[6] Black JA, Moland ES, Thomson KS (2005). AmpC disk test for detection of plasmid-mediated AmpC β-lactamases in Enterobacteriaceae lacking chromosomal AmpC beta-lactamases. J Clin Microbiol.

[7] Pérez-Pérez FJ, Hanson ND (2002). Detection of plasmid-mediated AmpC beta-lactamase genes in clinical isolates by using multiplex PCR. J Clin Microbiol.

[8] Song W, Moland ES, Hanson ND, Lewis JS, Jorgensen JH, Thomson KS (2005). Failure of cefepime therapy in treatment of Klebsiella Pneumoniae bacteremia. J Clin Microbiol.

[9] Steward CD, Rasheed JK, Hubert SK, Biddle JW, Raney PM, Anderson GJ (2001). Characterization of clinical isolates of Klebsiella Pneumoniae from 19 laboratories using the National Committee for clinical laboratory standards extended-spectrum beta-lactamase detection methods. J Clin Microbiol.

[10] Thomson KS (2001). Controversies about extended-spectrum and AmpC beta-lactamases. Emerg Infect Dis.

[11] CLSI (2010). Performance standards for antimicrobial susceptibility testing, M100-S20.

[12] Jarlier V, Nicolas MH, Fournier G, Philippon A. Extended broad-spectrum beta-lactamases conferring transferable resistance to newer beta-lactam agents in Enterobacteriaceae: hospital prevalence and susceptibility patterns. Rev Infect Dis. 10:867–878. http://www.ncbi.nlm.nih.gov/pubmed/3263690. Accessed 21 Feb 2017 10.1093/clinids/10.4.867 3263690

[13] Ingram PR, Inglis TJJ, Vanzetti TR, Henderson BA, Harnett GB, Murray RJ (2011). Comparison of methods for AmpC β-lactamase detection in enterobacteriaceae. J Med Microbiol.

[14] Cantarelli VV (2007). Inamine E, Brodt TCZ, Secchi C, Cavalcante BC, Pereira F de S. Utility of the ceftazidime-imipenem antagonism test (CIAT) to detect and confirm the presence of inducible AmpC beta-lactamases among enterobacteriaceae. Braz J Infect Dis.

[15] Philippon A, Arlet G, Jacoby GA (2002). Plasmid-determined AmpC-type beta-lactamases. Antimicrob Agents Chemother.

[16] Xiong Z, Li T, Xu Y, Li J (2007). Detection of CTX-M-14 extended-spectrum β-lactamase in Shigella sonnei isolates from China. J Inf Secur.

[17] Lonchel Magou C, Melin P, Gangoue-Pieboji J, Okomo Assoumou M, Boreux R, De Mol P (2013). Prevalence and spread of extended- spectrum b-lactamase-producing Enterobacteriaceae in Ngaoundere, Cameroon. Clin Microbiol Infect.

[18] Hanson ND (2003). AmpC β-lactamases: what do we need to know for the future?. J Antimicrob Chemother.

[19] Pitout JDD, Laupland KB (2008). Review extended-spectrum β-lactamase-producing Enterobacteriaceae: an emerging public-health concern. Lancet Infect Dis.

[20] Lahey Clinic. ß-Lactamase classification and amino acid sequences for TEM, SHV and OXA extended-Spectrum and inhibitor resistant enzymes. 2016. https://www.lahey.org/studies/.

[21] Le TMV, Baker S, Le TPT, Le TPT, Cao TT, Tran TTN (2009). High prevalence of plasmid-mediated quinolone resistance determinants in commensal members of the Enterobacteriaceae in ho chi Minh City, Vietnam. J Med Microbiol.

[22] Thomson N, Baker S, Pickard D, Fookes M, Anjum M, Hamlin N (2004). The role of prophage-like elements in the diversity of salmonella enterica serovars. J Mol Biol.

[23] Lau SM, Peng MY, Chang FY (2000). Outcomes of Aeromonas bacteremia in patients with different types of underlying disease. J Microbiol Immunol Infect.

[24] Llopis F, Grau I, Tubau F, Cisnal M, Pallares R (2004). Epidemiological and clinical characteristics of bacteraemia caused by Aeromonas spp. as compared with Escherichia Coli and Pseudomonas Aeruginosa. Scand J Infect Dis.

[25] Kollef MH (2008). Broad-Spectrum antimicrobials and the treatment of serious bacterial infections: getting it right up front. Clin Infect Dis.

[26] Kollef M (2003). Appropriate empirical antibacterial therapy for nosocomial infections: getting it right the first time. Drugs.

