# Supplementary material for: Phenotypic and genotypic characteristics of ESBL and AmpC producing organisms associated with bacteraemia in Ho Chi Minh City, Vietnam
Source: Antimicrob Resist Infect Control. 2017 Oct 16;6:105. doi: 10.1186/s13756-017-0265-1 (PMC5644090; doi:10.1186/s13756-017-0265-1)
Supplement: Supplementary file 1 — Representative results of the double disk diffusion test (A) for ESBL production and the AmpC disk test (B). Abbreviations. AMC: amoxicillin, CTX: cefotaxime, CAZ: ceftazidime, CRO: ceftriaxone, CLA: clavulanate, FEP: cefepime, FOX: cefoxitin, IPM: imipenem. (PDF 281 kb) [file 13756_2017_265_MOESM1_ESM.pdf]

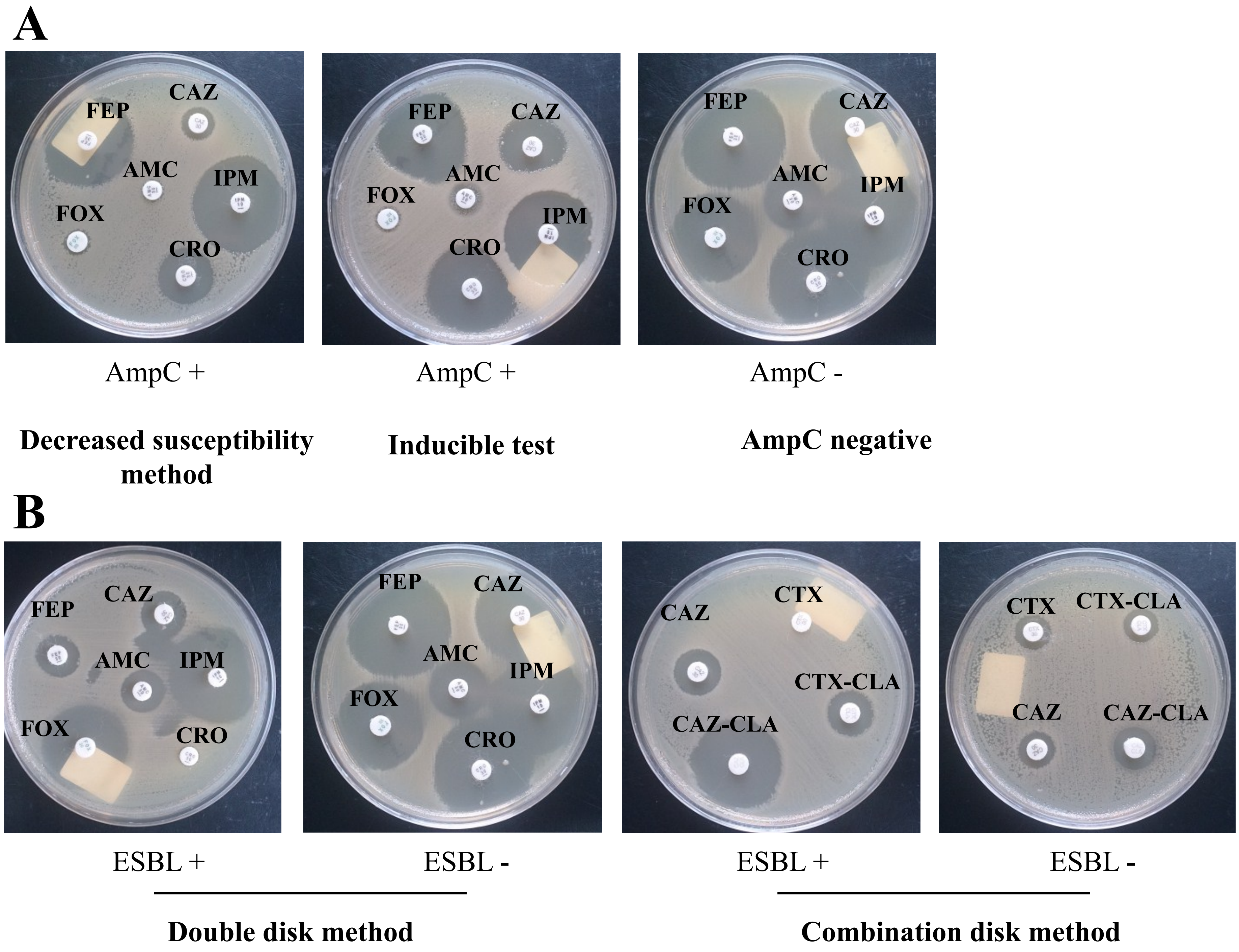
A
FEP
CAZ
AMC
IPM
FOX
CRO
AmpC +
AmpC +
AmpC -
Decreased susceptibility method
Inducible test
AmpC negative
B
CTX
CAZ-CLA
CTX-CLA
ESBL +
ESBL -
ESBL +
ESBL -
Double disk method
Combination disk method
