# Supplementary material for: Phenotypic and genotypic characteristics of ESBL and AmpC producing organisms associated with bacteraemia in Ho Chi Minh City, Vietnam
Source: Antimicrob Resist Infect Control. 2017 Oct 16;6:105. doi: 10.1186/s13756-017-0265-1 (PMC5644090; doi:10.1186/s13756-017-0265-1)
Supplement: Supplementary file 2 — The AmpC and ESBL primers used in this study. (DOCX 100 kb) [file 13756_2017_265_MOESM2_ESM.docx]

**Table S1.** **AmpC and ESBL primers used in this study.**

| Multiplex | Resistance phenotype | Gene | Primer name | Sequence (5’-3’) | Amplicon (bp) | Reference |
| --- | --- | --- | --- | --- | --- | --- |
| I | AmpC | MOX-1, MOX-2, CMY-1, CMY-8 -11 | MOXF^a^ | GCTGCTCAAGGAGCACAGGAT | 520 | [11] |
|  |  |  | MOXR^a^ | CACATTGACATAGGTGTGGTGC |  |  |
| I | AmpC | LAT-1 to LAT-4, CMY-2-7, BIL-1 | CITF | TGGCCAGAACTGACAGGCAAA | 462 | [11] |
|  |  |  | CITR | TTTCTCCTGAACGTGGCTGGC |  |  |
| I | AmpC | DHA-1, DHA-2 | DHAF | AACTTTCACAGGTGTGCTGGGT | 405 | [11] |
|  |  |  | DHAR | CCGTACGCATACTGGCTTTGC |  |  |
| I | AmpC | ACC | ACCF | AACAGCCTCAGCAGCCGGTTA | 346 | [11] |
|  |  |  | ACCR | TTCGCCGCAATCATCCCTAGC |  |  |
| I | AmpC | MIR-1T ACT-1 | EBCF | TCGGTAAAGCCGATGTTGCGG | 302 | [11] |
|  |  |  | EBCR | CTTCCACTGCGGCTGCCAGTT |  |  |
| I | AmpC | FOX-1 to FOX-5b | FOXF | AACATGGGGTATCAGGGAGATG | 190 | [11] |
|  |  |  | FOXR | CAAAGCGCGTAACCGGATTGG |  |  |
| II | ESBL | CTX-M1 | M1F | AAAAATCACTGCGCCAGTTC | 415 | [10] |
|  |  |  | M1R | AGCTTATTCATCGCCACGTT |  |  |
| II | ESBL | CTX-M2 | M2F | CGACGCTACCCCTGCTATT | 552 | [10] |
|  |  |  | M2R | CCAGCGTCAGATTTTTCAGG |  |  |
| II | ESBL | CTX-M9 | M9F | CAAAGAGAGTGCAACGGATG | 205 | [10] |
|  |  |  | M9R | ATTGGAAAGCGTTCATCACC |  |  |
| II | ESBL | CTX-M8/25 | M8F | CGCGTTAAGCGGATGATGC | 666 | [10] |
|  |  |  | M25F | GCACGATGACATTCGGG | 327 |  |
|  |  |  | M8/25R | AACCCACGATGTGGGTAGC |  |  |
| III | ESBL | TEM variants | TSO-T-F | TGCGGTATTATCCCGTGTTG | 296 | [12] |
|  |  |  | TSO-T-R | TCGTCGTTTGGTATGGCTTC |  |  |
| III | ESBL | SHV variants | TSO-S-F | AGCCGCTTGAGCAAATTAAAC | 713 | [12] |
|  |  |  | TSO-S-R | ATCCCGCAGATAAATCACCAC |  |  |
| III | ESBL | OXA-1, OXA-4-30 | TSO-O-F | GGCACCAGATTCAACTTTCAAG | 564 | [13] |
|  |  |  | TSO-O-R | GACCCCAAGTTTCCTGTAAGTG |  |  |
