# Supplementary material for: Phenotypic and genotypic characteristics of ESBL and AmpC producing organisms associated with bacteraemia in Ho Chi Minh City, Vietnam
Source: Antimicrob Resist Infect Control. 2017 Oct 16;6:105. doi: 10.1186/s13756-017-0265-1 (PMC5644090; doi:10.1186/s13756-017-0265-1)
Supplement: Supplementary file 3 — Summary of ESBL genes identified in 177 ESBL producing isolates. (DOCX 49 kb) [file 13756_2017_265_MOESM3_ESM.docx]

**Table S2: Summary of ESBL genes identified in 177 ESBL producing isolates.**

| ESBL Genes | | Number of isolates (Percent %) | |
| --- | --- | --- | --- |
| None detected | | 4 (2.3%) | 4 (2.3%) |
| Single gene | ***bla*_CTX-M_** | **63 (35.6%)** | 67 (37.8%) |
|  | *bla*_TEM_ | 2 (1.1%) |  |
|  | *bla*_SHV_ | 2 (1.1%) |  |
|  | *bla*_OXA_ | 0 (0%) |  |
| Multiple-genes | ***bla*_CTX-M_ and *bla*_TEM_** | **67 (37.9%)** | 106 (59.9%) |
|  | *bla*_CTX-M_ and *bla*_OXA_ | 27 (15.3%) |  |
|  | *bla*_CTX-M_ and *bla*_SHV_ | 2 (1.1%) |  |
|  | *bla*_TEM_ and *bla*_OXA_ | 1 (0.6%) |  |
|  | *bla*_CTX-M_ , *bla*_TEM_ and *bla*_OXA_ | 8 (4.5%) |  |
|  | *bla*_CTX-M_ , *bla*_TEM_ and *bla*_SHV_ | 1 (0.6%) |  |
| Total | | 177 (100%) | 177 (100%) |
