# Supplementary figures and images for: Phenotypic and genotypic characteristics of ESBL and AmpC producing organisms associated with bacteraemia in Ho Chi Minh City, Vietnam
Source: Antimicrob Resist Infect Control. 2017 Oct 16;6:105. doi: 10.1186/s13756-017-0265-1 (PMC5644090; doi:10.1186/s13756-017-0265-1)

**A**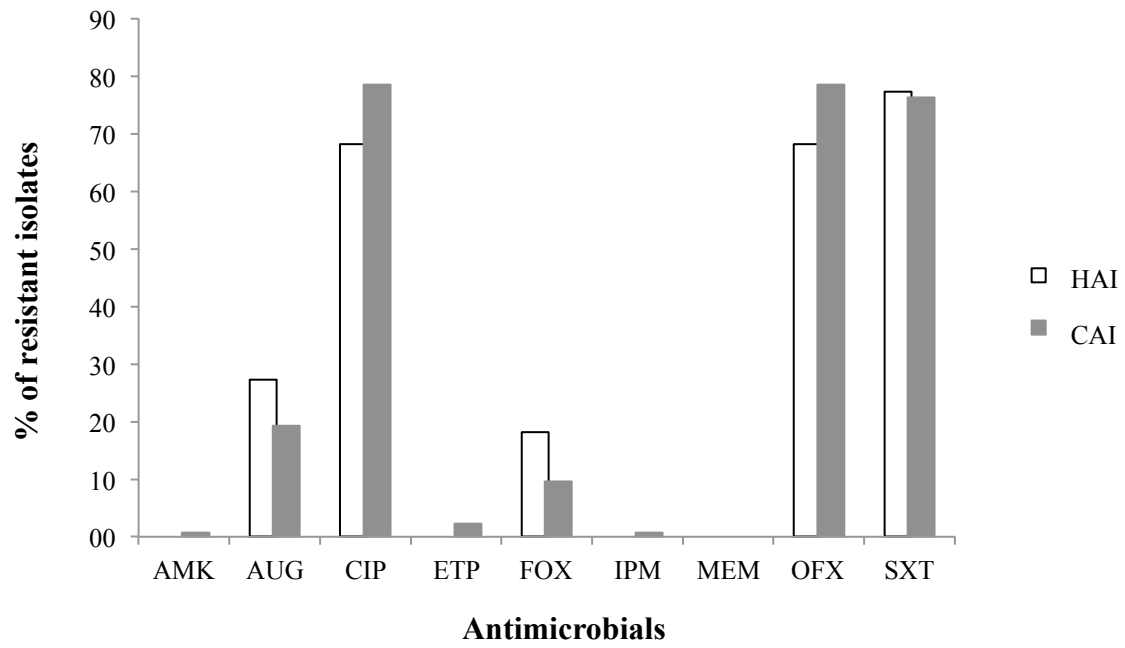**B**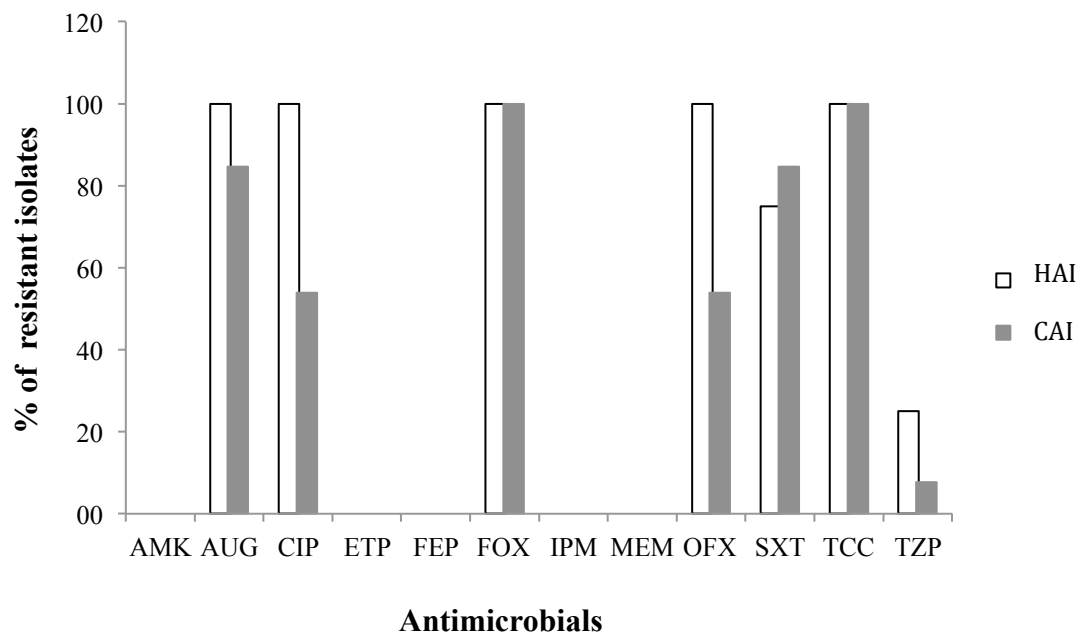

Supplement: Supplementary file 5 — Comparison of antimicrobial susceptibility profiles between CAI and HAI of the 177 ESBL (A) and 63 AmpC (B) producing E. coli. (A) The AMR phenotype of the ESBL producing E. coli (A) (n = 162) from CAI (n = 140) or HAI (n = 22) was scored for resistance to each drug and this was expressed as a percentage relative to the number of organisms in each group (CAI or HAI). (B) The AMR phenotype of the AmpC producing E. coli (n = 17) from CAI (n = 13) or HAI (n = 4) was scored for resistance to each drug and this was expressed as a percentage relative to the number of organisms in each group (CAI or HAI). No significant differences (p > 0.05) were found in antimicrobial resistance phenotype between CAI and HAI in either ESBL or AmpC producers. (PDF 47 kb) [file 13756_2017_265_MOESM5_ESM.pdf]
